# Genetic diversity among perennial wild rice *Oryza rufipogon* Griff., in the Mekong Delta

**DOI:** 10.1002/ece3.4978

**Published:** 2019-02-19

**Authors:** Dinh T. Lam, Bui C. Buu, Nguyen T. Lang, Kinya Toriyama, Ikuo Nakamura, Ryuji Ishikawa

**Affiliations:** ^1^ United Graduate School of Agricultural Science Iwate University Morioka Japan; ^2^ Institute of Agricultural Science for Southern Vietnam Ho Chi Minh City Vietnam; ^3^ High Agricultural Technology Research Institute (HATRI) Cantho Vietnam; ^4^ Graduate School of Agricultural Science Tohoku University Sendai Japan; ^5^ Graduate School of Horticulture Chiba University Matsudo Japan; ^6^ Faculty of Agriculture and Life Science Hirosaki University Hirosaki Japan

**Keywords:** clonal propagation, maternal lineage, Mekong Delta, mitochondrial rearrangement, *Oryza rufipogon*

## Abstract

*Oryza rufipogon* Griff. is a perennial species of wild rice widely distributed along the channels and rivers of the Mekong Delta, Vietnam. This study attempted to find centers of diversity among wild rice populations in this area and their inter‐relationships. The highest genetic diversity was found in the Dong Thap population and the lowest in the Can Tho population. Maternal diversity evaluated using chloroplast INDELs detected ten plastid types, five of which were novel relative to other Asian countries. The mitochondrial genome suggested two unique deletions. One 699‐bp deletion via short tandem repeats was accompanied by another deletion including *orf153*. All accessions carrying the mitochondrial type were found in a particular plastid type. This unique maternal lineage was confined to specific channels where it showed vigorous vegetative growth in comparison to upstream areas where various maternal lineages and maximum genetic diversity occurred. This area along the Mekong Delta is a center of not only nuclear but also maternal diversity.

## 1 INTRODUCTION

The wild species of genus *Oryza*is regarded as valuable resource for rice improvement because of its high genetic diversity (Brar, 2003; Sun, Wang, Li, Yoshimura, & Iwata, 2001). Application of wild rice to breeding programs can facilitate adaptation to climate change and meet the demand for food security in the face of rapid world population growth (Henry, 2016; Henry et al., 2010; Mickelbart, Hasegawa, & Bailey‐Serres, 2015; Moner et al., 2018).In this context, *Oryza rufipogon* and its relatives can provide a rich repository of genes and alleles for potential utilization in rice improvement with the help of genomics‐assisted breeding. Such studies can provide specific insight into natural genetic resources that can be preserved and utilized efficiently.

The wild rice species *Oryza rufipogon* Griff. is a common perennial known to be the progenitor of the Asian cultivated rice species, *O. sativa* L. (Oka, 1988; Vaughan, 1994). Many valuable genes conferring resistance to major biotic and abiotic stresses are being introduced into improved varieties (Brar & Khush, 1997; Ram, Majumder, Mishra, Ansari, & Padmavathi, 2007; Xiao et al., 1996; Yuan, Virmani, & Mao, 1989), for example, resistance to bacterial leaf blight (BB), brown plant hopper (BPH) and tungro virus, tolerance to aluminum toxicity, sulfate soil, and so on. Despite these advantages of wild rice, it is under serious threat and facing extinction due to ecological changes and human disturbance. Hence, effective conservation of this wild rice has become an urgent priority in many countries (Akimoto, Shimamoto, & Morishima, 1999; Gao, Zhang, Zhou, Gre, & Hong, 1996; Zhou, Chen, Wang, & Zhong, 1992).

The Mekong Delta, Vietnam, where the Mekong River flows out into the East Sea, has been considered a “biological treasure trove.” The delta shows high biodiversity of fauna and flora with 1,068 new species having been discovered (Fantz, 2008). According to the FAO database (FAOSTAT: http://www.fao.org/faostat/en/#home), the regional yield of paddy rice ranks 23rd in the world, but 6th in terms of production quantity, in view of the multiple cropping system made possible by the rich soil and abundant constantly available water resources. The area is also rich in wild rice species such as *O. rufipogon*, *O. nivara*, and *O. officinalis*. The delta is also the biggest rice granary in the country, playing a pivotal role in food security and accounting for more than 50% of total production, making Vietnam the second largest rice exporter in the world (Buu & Lang, 2007; Gephart, Blate, McQuistan, & Thompson, 2010; Ti et al., 2003). Because it is a rich source of not only wild rice species but also rice landraces, the Mekong Delta is considered to be one of the most important rice gene pools in the country (Buu, 1994, 1996; Xuan, 1975). The wild species of rice are widely distributed from upstream to downstream in the delta and their perennial nature makes them different from those in neighboring Cambodia (based on our field observation). Annual type is predominated around Phnom Penh area, whereas a few perennial populations were reported (Orn et al., 2015). Perennial type could be observed but became extinct because of the size and drastic infrastructure development. These wild rice are widespread along the river and channel systems in Mekong Delta, as well as occasionally in rice fields or marshes; particularly at Tram Chim sanctuary. Previous efforts at collaboration between Vietnamese and Japanese scientists to collect wild *Oryza*species, *O. rufipogon*, *O. nivara, O. officinalis*, and *O. granulate*, have been carried out in the delta (Buu & Lang, 1997, 2011). As a result, many useful accessions have been exploited for rice improvement over the last few decades, mostly to improve resistance to the brown plant hopper and blast, and tolerance to phosphorus deficiency, aluminum toxicity and acid sulfate soil (Buu & Lang, 2003; Nguyen et al., 2003). Although genetic variation of *O. rufipogon* in Vietnam has been studied, nucleus in genetic and maternal diversity has not yet been elucidated adequately (Cai, Wang, & Morishima, 2004; Ishii et al., 2011).

Molecular markers have provided a powerful tool for studies of genetic diversity among crop species (Gao, 2004; Olsen & Schaal, 2001; Song, Xu, Wang, Chen, & Lu, 2003), clarifying details of population structure and genetics, and having a significant impact on in situ conservation management (Barbier, 1989; Cai et al., 2004; Ishii et al., 2011; Kaewcheenchai et al., 2018; Wang et al., 2012). In fact, such data can be sampled efficiently from natural populations to monitor the transition of population structures in nature (Gao, 2004; Gao, Shaal, Zang, Jia, & Dong, 2002; Orn et al., 2015; Qian, Tianhua, Song, & Lu, 2005; Wang et al., 2012). Since the complete chloroplast (cp) genome became available, cytoplasmic molecular tools have also been developed to clarify evolutionary processes (Chen, Nakamura, Sato, & Nakai, 1993; Kano, Watanabe, Nakamura, & Hirai, 1993; Kim et al.., 2015; Masood et al., 2004; Sotowa et al., 2013; Takahashi, Sato, & Nakamura, 2008). Complete cp genomes are becoming easy to obtain by next‐generation sequencing and resequencing methodology (Wambugu, Brozynska, Furtado, Waters, & Henry, 2015; Waters, Nock, Ishikawa, Rice, & Henry, 2012).

The aims of the present study were to clarify (a) the genetic diversity of *O. rufipogon* in the Mekong Delta by using nuclear and cytoplasmic markers, and (b) their distribution along the delta based on maternal lineage. It was anticipated that the results would provide insight into the natural wild rice resources in this area that could be useful for biological conservation as well as exploitation in rice breeding programs.

## 2 MATERIALS AND METHODS

### 2.1 Field collection and plant materials

**Figure 1:**
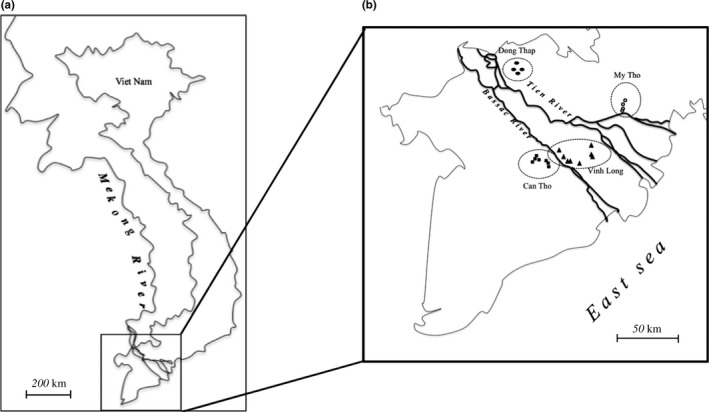
Collection sites for the wild rice *Oryza rufipogon* in the Mekong Delta, Vietnam. (a) Location of the Mekong Delta in southwest Vietnam. (b) Four populations of wild rice collected along the Mekong River, including Dong Thap as an upstream area, My Tho as a downstream area, Vinh Long as an intermediate area, and Can Tho as a flooding area. Dots and triangles indicate collection sites

Wild rice accessions were collected from the upstream to downstream reaches of the Mekong River in Vietnam during the period 2010–2015. The samples were classed into four distinct populations according to the geography and ecology of the Mekong river, as well as local expert opinions. These were named the Dong Thap population of 25 accessions, the Intermediate population of 59 accessions, the My Tho population of 27 accessions, and the Can Tho population of 55 accessions (Figure 1). Subpopulations were subsequently identified comprising several individuals corresponding to different collection sites. A maximum of eight individuals for each subpopulation were collected (Appendix Table A1).

A core collection derived from the National Bio‐Resource (NBR) Project in Japan (Nonomura et al., 2010) and 85 Thai wild rice accessions were also applied for verification (Kaewcheenchai et al., 2018). Additionally, one hundred wild stocks preserved at Cuu Long Rice Research Institute (CLRRI) were used to trace mitochondrial deletions in order to analyze mitochondrial variations from the past (Appendix Table A2).

### 2.2 Mitochondrial genome markers

A wild rice accession from the Can Tho population was subjected to next‐generation sequencing to obtain resequencing data against the mitochondrial genome. Details of the NGS protocol have been reported previously (Waters et al., 2012). Mt‐INDEL‐327994‐forward (agaatggtggaatctggtcaatctccatc) and mt‐INDEL‐329823‐reverse (attggatagtgatctcgggcacgagtgg) were used to detect one deletion. Nondeletion type of the PCR product would be 1,830 bp and deletion type 1,131 bp in size. Another presumed deletion included *orf153* in the mitochondrial genome. The presence or absence of PCR products was used to detect the *orf*153 deletion with *r*‐Taq (NEB Co. Ltd., Japan) using the primers *orf153f*:GTCTAGGGCTTCATCTTATGCC (forward) and CTAAGAAATCAGTAGAAATCGGG (reverse) which makes a 460 bp amplified product in Nipponbare mitochondrial genome (NC_011033).The PCR conditions were preheating at 94ºC for 3 min, 30 rounds at 94ºC for 10 s, 55ºC for 30 s and 72ºC for 30 s, and 72ºC for 5 min. The PCR products were then subjected to 0.8% agarose gel electrophoresis in 0.5 × TAE buffer because of the expected size of amplicons.

### 2.3 Molecular markers

Eight chloroplast INDELs (cpINDELs) developed in our previous study (Kaewcheenchai et al., 2018) were used to trace maternal lineages. Twenty nuclear SSR markers were applied to evaluate genetic diversity (Table 1). PCR products were amplified using a basic cycle of preheating at 94ºC for 3 min, followed by 30 rounds of 95ºC for 10 s, 55ºC for 30 s, and 72ºC for 30 s, and postheating at 72ºC for 5 min with Thermopol *Taq* polymerase (NEB Ltd., Japan). The amplified DNA fragments of both chloroplast and nuclear were mixed with a loading dye for electrophoresis on 6% denaturing polyacrylamide gel at 1,500 V for 2 hr in 0.5 × TBE buffer. The gels were then visualized by silver staining (Promega Co., Japan).

**Table 1:** Chloroplast INDEL markers and nuclear SSRs markers used in the study

Marker	Genome/Chromosome	Deletion or insertion sites/Genome position (bp)	Forward primer	Reverse primer
Chloroplast INDEL markers				
cpINDEL1	chloroplast	Deletion: 12670..12673	GGATTCACCGAAACAAACAACC	GCCAAATTGAGCAGGTTGCG
cpINDEL2	chloroplast	Deletion: 14012..14013	TTTGGGGAAGAAAACATCTTCC	TAAACGGAGAGAATCGACTAAG
cpINDEL3	chloroplast	Deletion: 17380..17385	AATTGCTCTCACCGCTCTTTC	TAGTCGAATTGTTGTATCAACTC
cpINDEL4	chloroplast	Deletion: 46087..46091	TAATTTGATATGGCTCGGACG	TGCTATGATTCTATGTTCTCC
cpINDEL5	chloroplast	Deletion: 46534..46539	AGATGGAGGAAATTGCACAAGG	CAAAACATGGATTTGGCTCAGG
cpINDEL8	chloroplast	Insertion: 57644^57645	TTTTACAGGAGTATCTAGTTGG	ATTACCTCTTTTTCGAGAACC
cpINDEL9	chloroplast	Insertion: 60865^60866	AAATCCTTTTAGGAGGGATTG	TCCACTACATCGCCTGAACC
cpINDEL12	chloroplast	Insertion: 77735^77736	TGTCTTTCCAGAAAGAAGAACC	TTGTTAAACCAGGTCGAATAC
Nuclear SSR markers				
RM3604	Chromosome 1	5140439	ATGTCAGACTCCGATCTGGG	TCTTGACCTTACCACCAGGC
RM8231	Chromosome 1	39927792	GCGTAAGATCTCCCTACCAC	CAACACATGATAGCACATGG
RM6853	Chromosome 2	8985893	CAACACGCACATCCTGTACC	CTCCAAAGACGAGACCAAGG
RM6301	Chromosome 3	2651356	CGCTACCTTATGCTGCTGTC	TCGGCTACAACCTCTCCTTC
RM5442	Chromosome 3	5528248	AGGAGACAGGAAAGCCTTCC	CGAGTCGACCAGGCTAGAAC
RM16262	Chromosome 4	178121	CTTTGACGCCCACCTTACTC	GCCCAGACTAGCATGATTGA
AL606650	Chromosome 4	31858308	CACATAGACCGAAATCGGGG	GACGGTAGGTAAAGTACAATC
RM146	Chromosome 5	18111333	CTATTATTCCCTAACCCCCATACCCTCC	AGAGCCACTGCCTGCAAGGCCC
RM8074	Chromosome 6	1415186	TACTACCACTTCTAGATGAGTTCAG	CTGAATACACTTCAATTTCTCTC
+29cat	Chromosome 6	30917713	CACGATCTAGAAGACGAGAG	CCAAATTACGCCTTCCTACC
RM214	Chromosome 7	13444643	CTGATGATAGAAACCTCTTCTC	AAGAACAGCTGACTTCACAA
RM284	Chromosome 8	21012219	ATCTCTGATACTCCATCCATCC	CCTGTACGTTGATCCGAAGC
RM1109	Chromosome 8	20353160	TCAAAATCACGTGTATGTAAGC	TTTACAAAGGACAGAGGGC
RM149	Chromosome 8	24724322	GCTGACCAACGAACCTAGGCCG	GTTGGAAGCCTTTCCTCGTAACACG
RM23805	Chromosome 9	5220232	GCATGCCCATCAACACTA	AGCGAGGACCAAATCCTTGT
RM3834	Chromosome 10	21951232	CTCGAGCTCCAACAAGAACC	GCTATGCTGAGCCGGAGTAG
RM311	Chromosome 10	9487243	TGGTAGTATAGGTACTAAACAT	TCCTATACACATACAAACATAC
RM5379	Chromosome 11	21796175	AGGGCATGCTTACATCCAAC	CATTTGCTTCTATGCCCCAG
RM309	Chromosome 12	21636510	GTAGATCACGCACCTTTCTGG	AGAAGGCCTCCGGTGAAG
RM6947	Chromosome 12	23974120	ATTAAACGTCCACTGCTGGC	GCTAGGTTAGTGGTGCAGGG

### 2.4 Data analysis

The data were subjected to principal component analysis using GenAlEx software (http://biology-assets.anu.edu.au/GenAlEx/Welcome.html). Genetic distances among accessions, the numbers of alleles (*N*_a_), observed heterozygosity (*H*_o_), and expected heterozygosity (*H*_e_) were calculated.

## 3 RESULTS

### 3.1 Chloroplast genome variations (maternal lineage)

In order to trace maternal lineages of wild rice along the Mekong river, eight chloroplast (cp) INDELs were genotyped. Only cpINDEL5 was monomorphic among all of the accessions collected in Vietnam. Remaining cpINDELs represented alternative genotypes except for cpINDEL3 carrying multiple alleles. These allelic combinations were used to identify different chloroplast types as plastid types (Appendix Table A3).

**Figure 2:**
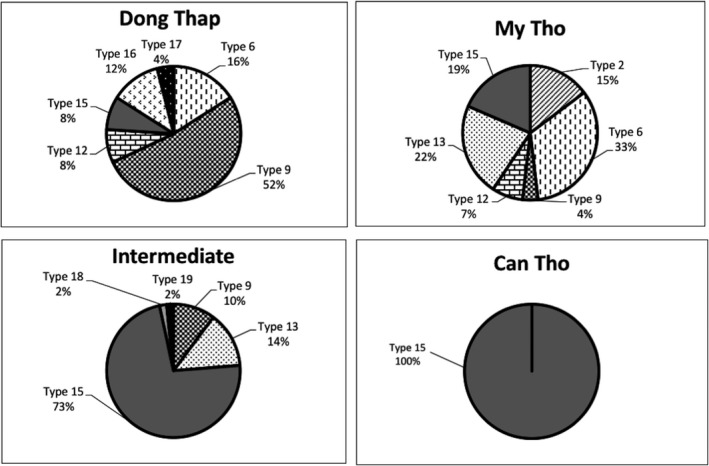
Compositions of maternal lineages among the Dong Thap, My Tho, and Intermediate populations

Among accessions from other South‐east Asian countries in the core collection and 85 wild rice accessions from Thailand, 19 plastid types were identified in total. The accessions in the core collection and Thai populations comprised six and 12 plastid types, respectively (Table 2). One hundred and sixty‐six accessions from Vietnam comprised ten plastid types, carrying five that were unique and different from the controls. These types were generated from Types 15 to 19, suggesting that wild rice in Vietnam had originated from distinct lineages. Type 15 was the most common in all regions, but was the only one present at Can Tho. The Dong Thap population was composed of three plastid types, Types 15, 16, and 17. The Intermediate population carried unique plastid types such as Types 18 and 19. The My Tho populations comprised six plastid types. Type 15 was found in all of the populations, but characterized canals at Can Tho, and also predominated in the Intermediate population (Figure 2).

**Table 2:** Frequency distribution of plastid types found in Vietnames wild rice, Thai wild rice, and NBR

Population	No. of accessions	No. of plastid types	Plastid types																		
			1	2	3	4	5	6	7	8	9	10	11	12	13	14	15	16	17	18	19
Control																					
NBR	32	6	–	–	4	1	20	–	–	–	2	2	–	–	3	–	–	–	–	–	–
Thailand^a^	85	12	1	1	43	–	11	2	2	2	7	–	1	2	1	12	–	–	–	–	–
Vietnamese wild rice																					
Dong Thap	25	6	–	–	–	–	–	4	–	–	13	–	–	2	–	–	2	3	1	–	–
Intermediate	59	5	–	–	–	–	–	–	–	–	6	–	–	–	8	–	43	–	–	1	1
My Tho	27	6	–	4	–	–	–	9	–	–	1	–	–	2	6	–	5	–	–	–	–
Can Tho	55	1	–	–	–	–	–	–	–	–	–	–	–	–	–	–	55	–	–	–	–

a Kaewcheenchai et al. (in press).

Although wild rice populations in Vietnam have been cataloged (Buu & Lang, 2011), maternal lineages have not yet been traced. Therefore, this result shows that perennial wild rice species in Vietnam have identical maternal lineages, thus, contributing to our understanding of the origin of maternal genetics.

### 3.2 Tracing maternal lineages with unique deletions in the mitochondrial genome

**Figure 3:**
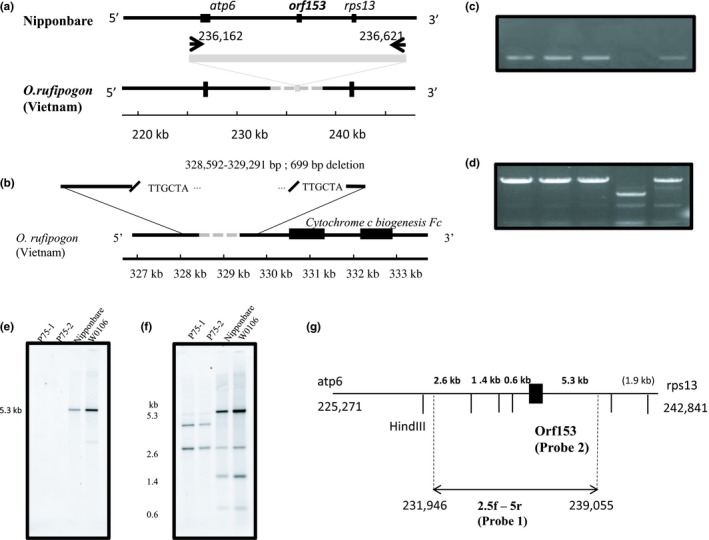
Unique deletions detected in the mitochondrial genome of wild rice, *Oryza rufipogon*, in the Mekong Delta. (a) Location of the presumed deletion around *orf153*. (b) Another deletion involving simple direct repeats. (c) INDEL pattern for the *orf153 deletion*. (d) INDEL pattern of the 669‐bp deletion. DNA templates were Nipponbare (lane 1), W0107, and W0108 for *O. rufipogon* originating in India (lanes 2 and 3). P75‐2 in the Can Tho population (lane 4).P36‐3 in the Intermediate population (lane 5). (d) INDEL pattern of the deletion spanning the bp 328,592 to bp 329,291 stretch. From left to right, Nipponbare, W0107, W0108, P75‐2, and P36‐3. (e) Southern blot showing the *orf153* deletion in the P75‐1 and P75‐2 wild rice accessions from the Can Tho population. (f) Southern blot showing higher rearrangement around *atp6* when probed with 2.5f‐5r (probe 1). (g) Location of Probe 1 as 2.5f‐5r between 231,946‐239,055nt in Nipponbare genome. Probe 2 is indicated as black box corresponding to *orf153*

Resequencing of the mitochondrial genome yielded two absence/deletion markers, which were confirmed by PCR amplification (Figure 3).One of these presumed deletions, termed the 699‐bp deletion, extending from bp 328, 592 to bp 329, 291 in the Nipponbare mitochondrial genome, was amplified and sequenced. It was flanked by tandem duplications of TTGCTA in Nipponbare. Using PCR amplificon, we also tried to confirm another presumed deletion that included *orf153*. However, this region was not amplified in a particular Vietnamese wild rice accession. The PCR products of *orf153* and its upstream region were used as probes to confirm the deletion by Southern hybridization. Specific Vietnamese accessions, P75‐1 and P75‐2, in the P75 subpopulation at Can Tho did not yield any signals. In order to clarify the mitochondrial rearrangement, a flanking probe was used. This showed that two accessions in the P75 subpopulation exhibited polymorphism relativeto Nipponbare and W0106, suggesting that a highly complex rearrangement may have deleted *orf153* in the P75 subpopulation.

**Figure 4:**
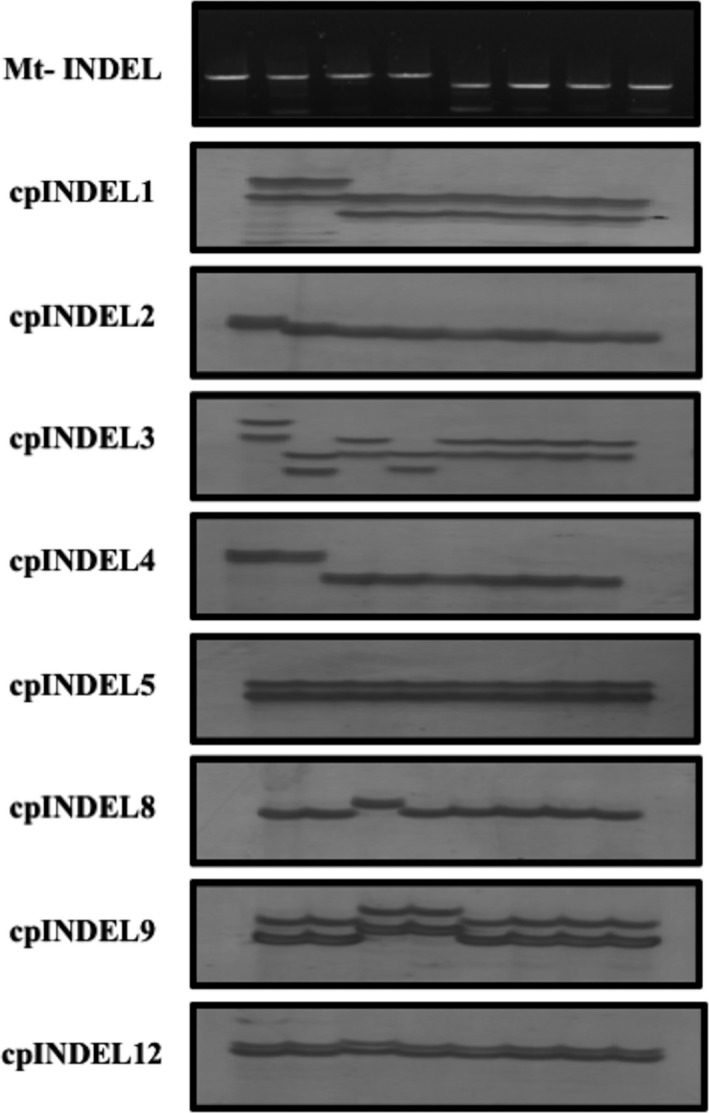
Banding pattern of cpINDEL and mitochondrial deletion showing accessions carrying the deletions is shared by an identical plastid type, Type 15. Template DNA from left to right: Nipponbare, P36‐3 (plastid type 9), P59‐2 (plastid type 2), P46‐4 (plastid type16), P75‐1, P36‐1, P53‐1, and P46‐2 belonging to plastid type 15. Markers from top downwards are listed beside each figure

All wild accessions from the Mekong Delta were screened for both mitochondrial INDEL markers, and this revealed that the two deletions were present in a single maternal lineage (Figure 4). This was a feature in all four populations and all accessions from the Can Tho population. In addition, only two accessions at Dong Thap shared these deletions but not in others in the CLRRI collection (Table 3). All accessions carrying the deletions corresponded to a particular plastid type, Type 15.

**Table 3:** A frequency of a mitochondrial deletion (327,994–329,823 bp)accompanied with a particular plastid type

Population	No. of accessions	mt‐Deletion			Plastid type
		Nondeletion	Deletion	(%)	
CLRRI's Genbank					
Hau giang	4	4	0	0	–
Dong Thap	71	69	2	3	Type 15^a^
Long An	25	25	0	0	–
Natural habitat					
Dong Thap	25	23	2	8	Type 15
Intermediate area	59	16	43	73	Type 15
My Tho	27	22	5	19	Type 15
Can Tho	55	0	55	100	Type 15

a Plastid type of accessions carrying the mitochondrial deletion.

### 3.3 Genetic diversity and phylogenetic relationships evaluated using nuclear SSR markers

Genetic diversity was estimated using 20 SSR markers (Table 4). The highest number of alleles was found in RM3834 (*N*_a_ = 8), and the lowest in RM6947 (*N*_a_ = 2) in the overall population. The observed heterozygosity (*H*_o_) ranged from 0.054 to 1.000 among the 20 loci and from 0.60 to 0.644 among the populations. The Dong Thap population showed the highest diversity, *H*_e_ = 0.659, whereas the Can Tho population showed the lowest at *H*_e_ = 0.300. The *H*_e_ scores for the My Tho and Intermediate populations were *H*_e_ = 0.613 and *H*_e_ = 0.557, respectively. All accessions in the seven subpopulations from Can Tho shared the single genotypes over 20 loci. Twelve of the 20 examined loci were heterozygous, and the remaining eight were monomorphic. Vegetative propagation was inferred from the genotypes. The same genotypes were not found among other materials examined.

**Table 4:** Genetic diversity among four wild populations in Mekong Delta evaluated by 20 SSR markers

Locus	Can Tho			Intermediate			Dong Thap			My Tho			Overall population		
	*N*_a_	*H*_o_	*H*_e_	*N*_a_	*H*_o_	*H*_e_	*N*_a_	*H*_o_	*H*_e_	*N*_a_	*H*_o_	*H*_e_	*N*_a_	*H*_o_	*H*_e_
RM3604	2	1.000	0.500	6	0.814	0.729	9	0.520	0.745	3	0.481	0.615	6	0.549	0.654
AL606650	2	1.000	0.500	5	0.729	0.719	9	0.680	0.830	6	0.556	0.712	6	0.757	0.697
RM311	1	0.000	0.000	5	0.271	0.699	4	0.280	0.593	4	0.185	0.644	4	0.224	0.489
+29CAT	2	1.000	0.500	6	1.000	0.705	6	0.840	0.777	5	0.963	0.787	5	0.824	0.661
RM8074	1	0.000	0.000	4	0.305	0.689	4	0.800	0.678	4	0.815	0.623	4	0.497	0.522
RM5379	2	1.000	0.500	6	1.000	0.787	7	0.800	0.757	6	1.000	0.679	6	0.950	0.713
RM8231	1	0.000	0.000	2	0.136	0.126	6	0.600	0.714	5	0.519	0.652	4	0.306	0.366
RM146	1	0.000	0.000	2	0.034	0.033	4	0.440	0.410	3	0.667	0.483	3	0.290	0.339
RM16262	1	0.000	0.000	6	0.661	0.522	5	0.680	0.518	3	1.000	0.575	4	0.627	0.444
RM214	2	1.000	0.500	7	1.000	0.700	7	0.840	0.773	6	0.963	0.750	6	0.951	0.698
RM284	2	1.000	0.500	4	0.746	0.618	6	0.680	0.734	5	1.000	0.634	5	0.847	0.641
RM1109	2	1.000	0.500	5	1.000	0.662	7	0.640	0.774	6	1.000	0.679	5	0.910	0.668
RM149	2	1.000	0.500	8	1.000	0.838	7	0.840	0.755	7	1.000	0.795	7	0.960	0.741
RM23805	2	1.000	0.500	3	0.339	0.521	4	0.320	0.570	3	0.000	0.535	3	0.373	0.531
RM3834	2	1.000	0.500	9	1.000	0.790	10	1.000	0.854	8	1.000	0.818	8	1.000	0.764
RM309	2	1.000	0.500	3	0.610	0.576	6	1.000	0.783	6	0.519	0.703	4	0.780	0.638
RM6947	1	0.000	0.000	2	0.136	0.126	2	0.000	0.077	1	0.000	0.000	2	0.054	0.067
RM6301	1	0.000	0.000	3	0.322	0.277	3	0.240	0.339	2	0.481	0.366	3	0.281	0.266
RM6853	1	0.000	0.000	3	0.288	0.289	4	0.680	0.663	4	0.222	0.593	3	0.312	0.409
RM5442	2	1.000	0.500	6	0.847	0.736	10	0.920	0.827	4	0.519	0.608	6	0.799	0.686
Mean	1.6	0.600	0.300	4.8	0.612	0.557	6.0	0.640	0.659	4.6	0.644	0.613	4.4	0.615	0.550
*SE*	0.0	0.042	0.021	0.5	0.078	0.055	0.5	0.060	0.044	0.4	0.078	0.040	0.2	0.039	0.026

**Figure 5:**
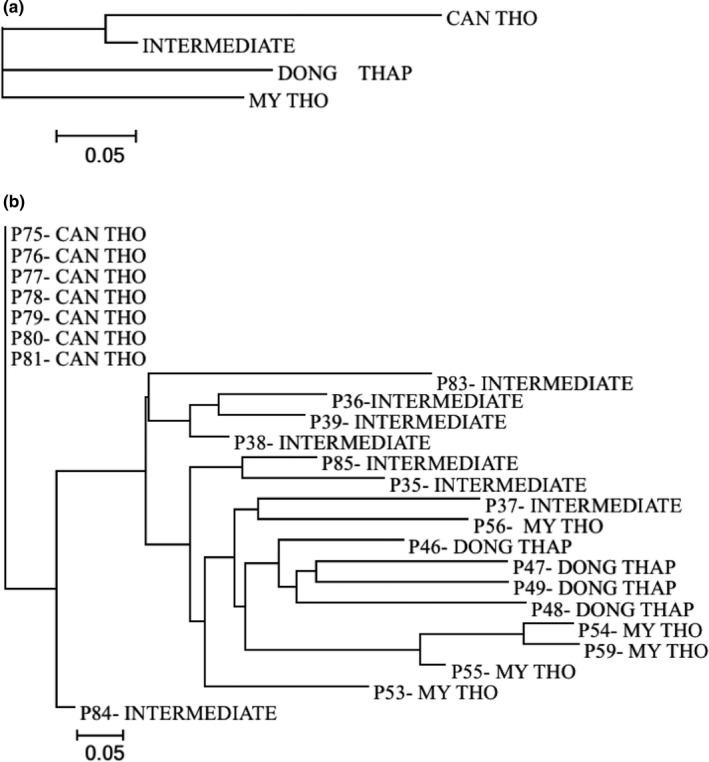
Phylogenetic tree of Vietnamese wild rice *Oryza rufipogon* categorized using 20 SSR markers. (a) Phylogenetic tree between four populations. (b) Phylogenetic tree among all subpopulations

A phylogenetic tree was constructed based on a distance matrix generated with the 20 SSR markers (Figure 5). As the four populations formed different clades, subpopulations making up each population were applied. There were two distinct clades: one including Dong Thap, Intermediate, and My Tho subpopulations, and the other including the Can Tho population. This suggested that wild rice at Can Tho is unique in comparison to the others.

## 4 DISCUSSION

Previous studies have attempted to characterize and exploit wild rice species in the Mekong Delta without investigating their origin, or clarifying genetic variations among them (Buu, 1996; Buu & Lang, 2007; Lang et al., 2012). The present study focused on genetic variation in *O. rufipogon* specimen collection along the Mekong River and attempted to know how they distribute along the river system by using maternally inherited markers. The unique genetic resource in *O. rufipogon* in the Mekong Delta yielded five novel plastid types, among which Type 15 was accompanied by a unique mitochondrial lineage showing marked rearrangement of the mitochondrial genome. The maternal lineage might have arisen upstream of the delta and become dispersed into the downstream area. The maternal lineage at Dong Thap, however, did not share the same nuclear genotype as those at Can Tho. One descendant belonging to the maternal lineage may have had the ability to form clones and occupy particular channels. Highly vegetative propagation and migration by drifting across canals may also have affected the structure of the Can Tho populations. This is the unique nature of wild rice inhabited along the Mekong Delta.

In contrast to the unique population in Can Tho, high genetic variation was found in the upstream area, at Dong Thap. This higher variation allowed a breeding program that successfully created AS996, carrying higher acid sulfate tolerance (Can & Lang, 2007; Khush & Virk, 2005; Lang et al., 2012). This higher degree of diversity might be due to ecological factors that have a great influence on genetic differentiation among wild rice populations (Orn et al., 2015). In fact, the Dong Thap wild population is distributed widely in a government conservation area at Tram Chim National Park. The process of conserving wild populations under natural conditions has been done accomplished with minimal human disturbance, thus, helping to maintain higher genetic diversity. As water flow may distribute individual plants downstream, areas en route such as the My Tho and Intermediate areas between My Tho and Can Tho may maintain relatively diverse variation, compared to channels at Can Tho. Currently, efforts to collect wild *Oryza* species are suspended in Vietnam, although accessions have been exploited for breeding programs to some extent. Meanwhile, wild populations have been seriously threatened and faced with extinction due to infrastructure development. Many subpopulations investigated in this study have been severely degraded by human disturbance; at least one wild subpopulation at site P78 has completely disappeared because of road construction. Although several wild species have been preserved at the CLRRI gene bank for ex‐situ conservation, the entire range of genetic variation has not been covered. Therefore, effective conservation management for *O. rufipogon* in upstream areas such as Dong Thap is becoming even more of an urgent priority. Our assessment of the genetic diversity would be available to collect valuable resources efficiently before they would be extinct.

## CONFLICT OF INTEREST

None declared.

## AUTHOR CONTRIBUTIONS

DTL and BCB managed core collection of perennial wild rice. DTL, BCB, NTL, IN, KT, and RI surveyed natural populations. DTL contributed to analyze with molecular markers. RI set up markers and genome analysis.

## Data Availability

All data were included in this manuscript.

## APPENDIX 1

**Table A1:** GPS records of wild rice subpopulations examined in this study

Population	Year of surveys	GPS point	Habitant	Region	No. of accessions
P75	2010	N9 59.384 E105 39.688	Along a particular canal in Bassac River west side	Can Tho	8
P76	2010	N9 59.431 E105 39.462	Along a particular canal in Bassac River west side	Can Tho	8
P77	2010	N9 59.115 E105 39.014	Along a particular canal in Bassac River west side	Can Tho	8
P78	2010	N9 59.855 E105 40.365	Along a particular canal in Bassac River west side	Can Tho	7
P79	2010	N9 57.783 E105 44.896	Along a particular canal in Bassac River west side	Can Tho	8
P80	2010	N9 57.342 E105 45.752	Flood plains nearby a canal in Bassac River west side	Can Tho	8
P81	2010	N9 56.484 E105 46.042	Flood plains nearby a canal in Bassac River west side	Can Tho	8
P83	2010	N10 02.731 E105 50.148	Between Bassac river and Tien River	Vinh Long	8
P84	2010	N9 59.718 E105 52.890	Between Bassac river and Tien River	Vinh Long	6
P85	2010	N9 58.219 E105 55.506	Between Bassac river and Tien River	Vinh Long	8
P35	2014	N09 58 13.14 E105 55 30.64	Between Bassac river and Tien River	Vinh Long	7
P36	2014	N09 57 09.36 E105 59 57.58	Between Bassac river and Tien River	Vinh Long	8
P37	2014	N10 00 14.32 E106 06 04.61	Between Bassac river and Tien River	Vinh Long	8
P38	2014	N10 00 12.10 E106 06 03.65	Between Bassac river and Tien River	Vinh Long	8
P39	2014	N10 05 30.83 E106 05 40.22	Between Bassac river and Tien River	Vinh Long	6
P46	2015	N10 42 22.69 E105 32 23.23	Tram Chim Sanctuary for in‐situ conservation	Dong Thap Muoi	8
P47	2015	N10 43 00.19 E105 30 04.59	Tram Chim Sanctuary for in‐situ conservation	Dong Thap Muoi	8
P48	2015	N10 43 04.95 E105 30 01.84	Tram Chim Sanctuary for in‐situ conservation	Dong Thap Muoi	8
P49	2015	N10 41 36.04 E105 31 37.86	Tram Chim Sanctuary for in‐situ conservation	Dong Thap Muoi	1
P53	2015	N10 23 43.91 E106 20 14.81	Road side near by Tien River	My Tho	3
P54	2015	N10 23 43.88 E106 20 14.81	Road side near by Tien River	My Tho	7
P55	2015	N10 23 48.25 E106 20 15.39	Road side near by Tien River	My Tho	5
P56	2015	N10 23 48.25 E106 20 15.39	Road side near by Tien River	My Tho	8
P59	2015	N10 25 13.91 E106 20 24.84	Road side near by Tien River	My Tho	4

**Table A2:** Wild rice accessions conserve in Cuu Long Rice Research Institute 's Genbank, and NBR wild rice accessions examined in this experiment

Accessions	Current conserved	Origin
1	CLRRI Genebank	Dong Thap Province
2	CLRRI Genebank	Dong Thap Province
3	CLRRI Genebank	Dong Thap Province
4	CLRRI Genebank	Dong Thap Province
5	CLRRI Genebank	Dong Thap Province
6	CLRRI Genebank	Dong Thap Province
7	CLRRI Genebank	Dong Thap Province
8	CLRRI Genebank	Dong Thap Province
9	CLRRI Genebank	Dong Thap Province
10	CLRRI Genebank	Dong Thap Province
11	CLRRI Genebank	Dong Thap Province
12	CLRRI Genebank	Dong Thap Province
13	CLRRI Genebank	Hau giang Province
14	CLRRI Genebank	Hau giang Province
15	CLRRI Genebank	Hau giang Province
16	CLRRI Genebank	Hau giang Province
17	CLRRI Genebank	Dong Thap Province
18	CLRRI Genebank	Dong Thap Province
19	CLRRI Genebank	Dong Thap Province
20	CLRRI Genebank	Dong Thap Province
21	CLRRI Genebank	Dong Thap Province
22	CLRRI Genebank	Dong Thap Province
23	CLRRI Genebank	Dong Thap Province
24	CLRRI Genebank	Dong Thap Province
25	CLRRI Genebank	Dong Thap Province
26	CLRRI Genebank	Dong Thap Province
27	CLRRI Genebank	Dong Thap Province
28	CLRRI Genebank	Dong Thap Province
29	CLRRI Genebank	Dong Thap Province
30	CLRRI Genebank	Dong Thap Province
31	CLRRI Genebank	Dong Thap Province
32	CLRRI Genebank	Dong Thap Province
33	CLRRI Genebank	Dong Thap Province
34	CLRRI Genebank	Dong Thap Province
35	CLRRI Genebank	Dong Thap Province
36	CLRRI Genebank	Dong Thap Province
37	CLRRI Genebank	Dong Thap Province
38	CLRRI Genebank	Dong Thap Province
39	CLRRI Genebank	Dong Thap Province
40	CLRRI Genebank	Dong Thap Province
41	CLRRI Genebank	Dong Thap Province
42	CLRRI Genebank	Dong Thap Province
43	CLRRI Genebank	Dong Thap Province
44	CLRRI Genebank	Dong Thap Province
45	CLRRI Genebank	Dong Thap Province
46	CLRRI Genebank	Dong Thap Province
47	CLRRI Genebank	Dong Thap Province
48	CLRRI Genebank	Dong Thap Province
49	CLRRI Genebank	Dong Thap Province
50	CLRRI Genebank	Dong Thap Province
51	CLRRI Genebank	Dong Thap Province
52	CLRRI Genebank	Dong Thap Province
53	CLRRI Genebank	Dong Thap Province
54	CLRRI Genebank	Dong Thap Province
55	CLRRI Genebank	Dong Thap Province
56	CLRRI Genebank	Dong Thap Province
57	CLRRI Genebank	Dong Thap Province
58	CLRRI Genebank	Dong Thap Province
59	CLRRI Genebank	Dong Thap Province
60	CLRRI Genebank	Dong Thap Province
61	CLRRI Genebank	Dong Thap Province
62	CLRRI Genebank	Dong Thap Province
63	CLRRI Genebank	Dong Thap Province
64	CLRRI Genebank	Dong Thap Province
65	CLRRI Genebank	Dong Thap Province
66	CLRRI Genebank	Dong Thap Province
67	CLRRI Genebank	Dong Thap Province
68	CLRRI Genebank	Dong Thap Province
69	CLRRI Genebank	Dong Thap Province
70	CLRRI Genebank	Dong Thap Province
71	CLRRI Genebank	Dong Thap Province
72	CLRRI Genebank	Dong Thap Province
73	CLRRI Genebank	Dong Thap Province
74	CLRRI Genebank	Dong Thap Province
75	CLRRI Genebank	Dong Thap Province
76	CLRRI Genebank	Long An Province
77	CLRRI Genebank	Long An Province
78	CLRRI Genebank	Long An Province
79	CLRRI Genebank	Long An Province
80	CLRRI Genebank	Long An Province
81	CLRRI Genebank	Long An Province
82	CLRRI Genebank	Long An Province
83	CLRRI Genebank	Long An Province
84	CLRRI Genebank	Long An Province
85	CLRRI Genebank	Long An Province
86	CLRRI Genebank	Long An Province
87	CLRRI Genebank	Long An Province
88	CLRRI Genebank	Long An Province
89	CLRRI Genebank	Long An Province
90	CLRRI Genebank	Long An Province
91	CLRRI Genebank	Long An Province
92	CLRRI Genebank	Long An Province
93	CLRRI Genebank	Long An Province
94	CLRRI Genebank	Long An Province
95	CLRRI Genebank	Long An Province
96	CLRRI Genebank	Long An Province
97	CLRRI Genebank	Long An Province
98	CLRRI Genebank	Long An Province
99	CLRRI Genebank	Long An Province
100	CLRRI Genebank	Long An Province
W0106	National Bio‐Resource, Japan	Phulankara, near Cuttack, Orissa, India
W0107	National Bio‐Resource, Japan	Pahala, Orissa, India
W0108	National Bio‐Resource, Japan	Cuttack, Orissa, India
W0120	National Bio‐Resource, Japan	Cuttack, Orissa, India
W0137	National Bio‐Resource, Japan	Kadiam, Andhra, India
W0180	National Bio‐Resource, Japan	Ngao, Lamphang, Thailand
W0593	National Bio‐Resource, Japan	Binjai Rendah, Malasia
W0610	National Bio‐Resource, Japan	Myanmar
W0630	National Bio‐Resource, Japan	Myanmar
W1294	National Bio‐Resource, Japan	Musuan, Mindanao, Philippines
W1551	National Bio‐Resource, Japan	Saraburi, Thailand
W1666	National Bio‐Resource, Japan	Siliguri, India
W1669	National Bio‐Resource, Japan	Orissa, India
W1681	National Bio‐Resource, Japan	Orissa, India
W1685	National Bio‐Resource, Japan	Orissa, India
W1690	National Bio‐Resource, Japan	Chiengrai, Thailand
W1715	National Bio‐Resource, Japan	China
W1807	National Bio‐Resource, Japan	Sri Lanka
W1852	National Bio‐Resource, Japan	Chiang Saen, Thailand
W1865	National Bio‐Resource, Japan	Saraburi, Thailand
W1866	National Bio‐Resource, Japan	Saraburi, Thailand
W1921	National Bio‐Resource, Japan	Saraburi, Thailand
W1939	National Bio‐Resource, Japan	Bangkoknoi, Thailand
W1945	National Bio‐Resource, Japan	No description
W1981	National Bio‐Resource, Japan	Palembang, Indonesia
W2003	National Bio‐Resource, Japan	from Pajani to Bombay, India
W2014	National Bio‐Resource, Japan	India
W2051	National Bio‐Resource, Japan	Hobiganji, Bangladesh
W2263	National Bio‐Resource, Japan	Cambodia
W2265	National Bio‐Resource, Japan	Laos
W2266	National Bio‐Resource, Japan	Laos
W2267	National Bio‐Resource, Japan	Laos

**Table A3:** Defination of plastid types based on genotype of eight chloroplast INDEL markers

Locus	Plastid types^a^																		
	1	2	3	4	5	6	7	8	9	10	11	12	13	14	15	16	17	18	19
cpINDEL1	1	1	1	1	1	1	1	1	2	2	2	2	2	2	1	1	1	1	1
cpINDEL2	1	1	1	1	1	1	2	2	1	1	2	2	2	2	1	1	1	1	1
cpINDEL3	1	1	2	2	2	2	2	2	1	2	1	1	2	3	1	1	1	−1	−1
cpINDEL4	1	1	1	1	1	1	1	1	2	2	1	2	2	2	1	1	1	1	1
cpINDEL5	1	2	1	2	2	2	1	2	2	2	2	2	2	2	2	2	2	2	2
cpINDEL8	2	2	2	1	2	2	2	2	1	1	1	1	1	1	1	1	2	2	2
cpINDEL9	2	2	2	2	2	2	2	2	1	1	1	1	1	1	1	2	2	2	2
cpINDEL12	2	1	2	1	1	2	2	1	1	1	1	1	1	1	1	1	2	1	2

a Type 1 ~ 14 were detected in a core collection (NBR) and Thai wild rice populations (data not published). Allele numbers were given when compared between Nipponbare and Thai45‐2 accession. Smaller PCR product was given allele 1 and larger one allele 2. When more shorter fragment was amplified, then allele −1 was given.
